# Primary Hyperparathyroidism Masquerading as Rickets: Diagnostic Challenge and Treatment Outcomes

**DOI:** 10.4274/Jcrpe.1060

**Published:** 2013-12-12

**Authors:** Deep Dutta, Manoj Kumar, Ram Narayan Das, Saumik Datta, Dibakar Biswas, Sujoy Ghosh, Satinath Mukhopadhyay, Subhankar Chowdhury

**Affiliations:** 1 IPGMER & SSKM Hospital, Department of Endocrinology & Metabolism, Calcutta, India; 2 IPGMER & SSKM Hospital, Department of Pathology, Calcutta, India

**Keywords:** primary hyperparathyroidism, rickets, Parathyroid adenoma

## Abstract

Primary hyperparathyroidism (PHPT) is extremely uncommon among children and is more likely to be associated with genetic syndromes, multiglandular involvement, and more severe symptoms. Rickets can very rarely be the presenting feature of PHPT in children. Rickets was diagnosed in a 12-year-old girl presenting with short stature, genu valgum, eversion deformity at the ankle joints, and flat feet. Radiograms showed generalized osteopenia, widening of the distal ends of the long bones along with splaying, cupping and fraying. Biochemical evaluation revealed low serum calcium (7.8 mg/dL), low phosphorus (1.4 mg/dL), vitamin-D deficiency [25-hydroxy-vitamin-D (25(OH)D): 8.7 ng/mL], and elevated intact parathyroid hormone (PTH, 811 pg/mL). Re-evaluation due to lack of clinical improvement following vitamin-D and calcium supplementation revealed hypercalcemia 11.9 mg/dL, normal 25(OH)D 41 ng/mL, persistence of elevated PTH 632 pg/mL. A 99mTc-sestamibi scan showed increased uptake at the lower pole of the right lobe of the thyroid. A right inferior parathyroidectomy was performed. Histopathology revealed chief cell type parathyroid adenoma. Last evaluated 4 months after surgery, the bone pains and proximal weakness had resolved, with significant improvement in the patient’s quality of life. Rickets in the setting of PHPT often masks the classical phenotype of PHPT. In a child with rickets, lack of improvement following vitamin-D supplementation, hypercalcemia at presentation or following vitamin-D supplementation are warning signs which necessitate further evaluation to rule out PHPT.

**Conflict of interest:**None declared.

## INTRODUCTION

Primary hyperparathyroidism (PHPT) most commonly presents in the fifth and sixth decade of life, especially among post-menopausal women. It is extremely uncommon among children (1). PHPT in children is more likely to be symptomatic. However, pediatric cases of PHPT most commonly present as asymptomatic hypercalcemia in the Western countries, in contrast to India where a majority of young patients present with hypercalcemia and its complications (nephrolithiasis, fractures, and acute pancreatitis), multiglandular involvement, or with associated genetic syndromes (2,3). Rickets as the presenting feature of PHPT in children is extremely rare with less than 19 cases reported to date (3,4). Here, we report a 12-year-old girl presenting with typical features of rickets, diagnosed to have PHPT due to a right inferior parathyroid adenoma and who showed resolution of all clinical features following parathyroidectomy.

## CASE REPORT

A 12-year-old girl with a normal perinatal history and milestones presented with short stature, lack of development of secondary sexual features, progressively worsening leg deformities and walking difficulty, symptoms which had appeared at age 8 years along with bone pains. Proximal weakness was also noted in the past 1 year. The patient mostly stayed indoors due to her limited physical activity, thus had also limited sun exposure.

**Figure 1:**
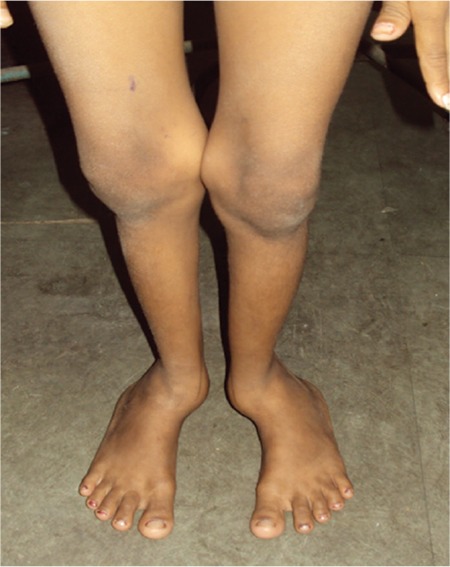
The legs of the patient showing muscle wasting, knock knees, bilateral eversion deformity at ankle joints, and flat feet

**Figure 2:**
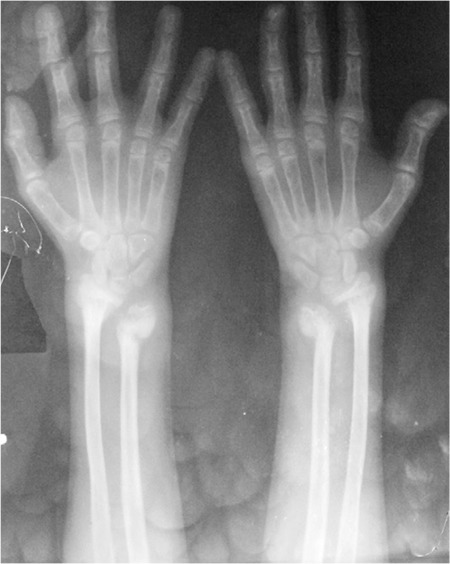
Radiograph of the hands and forearms showing generalized osteopenia, widening of the distal ends of the radius and ulna along with cupping, fraying and splaying of the metaphyses

**Figure 3:**
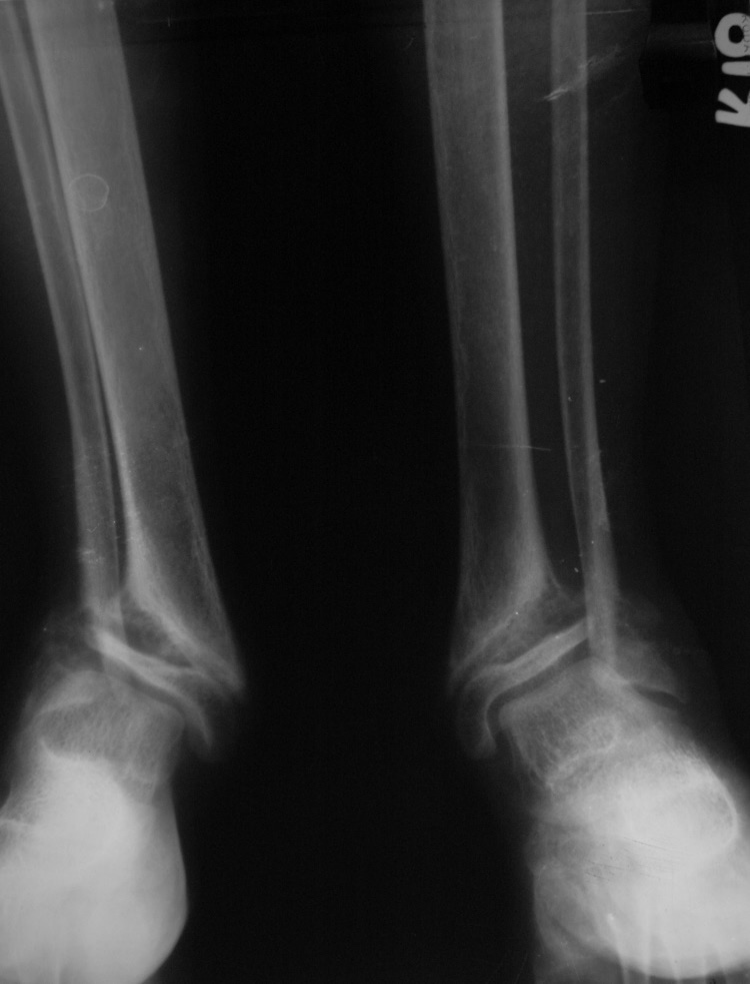
Radiograph of the ankle joints showing widening of the lower end of tibia, splaying and fraying of the metaphyses along with eversion deformity of the ankle joints

**Figure 4:**
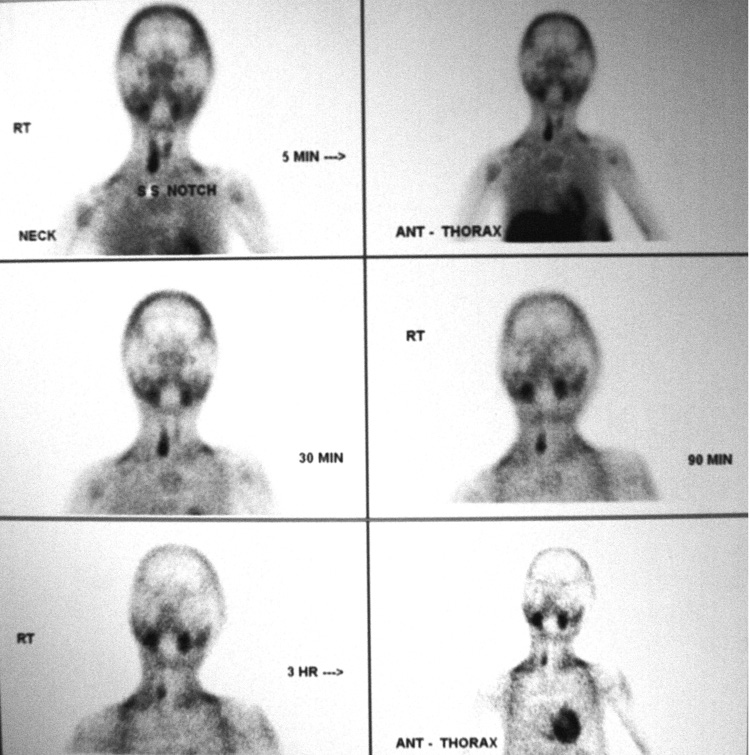
99mTc-sestamibi parathyroid scan revealing increased uptake in the right inferior thyroid region, a finding which persisted on delayed imaging at 90 minutes and 3 hours

The patient had significant short stature [height 132.5 cm; standard deviation score (SDS): -2.17; target height SDS: -0.22], widening of wrists, genu valgum, bilateral eversion deformity at the ankle joints along with flat feet, generalized muscle wasting (more prominent in the lower limb), and proximal muscle weakness (Figure 1). She was prepubertal by Tanner sexual maturity staging. Skeletal radiography revealed generalized osteopenia, widening of the distal ends of the long bones (radius, ulna, femur, and tibia) along with splaying, cupping and fraying (Figures 2, 3). Results of biochemical evaluation are given in Table 1 and were suggestive of vitamin D-deficiency rickets. She received weekly sachets of 60 000 U of cholecalciferol (DRISE, USV, Mumbai, India) for 6 weeks, and thereafter monthly along with calcium carbonate tablets containing 500 mg of elemental calcium per tablet (Shelcal, Elder, Mumbai, India). There was no improvement in the bone pains and proximal weakness. Re-evaluation at 16 weeks revealed hypercalcemia, worsening of the hypophosphatemia, persistence of the elevated parathyroid hormone (PTH) along with normalization of serum 25-hydroxy-vitamin-D [25(OH)D] (Table 1). 99mTc-sestamibi scan revealed increased uptake at the lower pole of the right lobe of the thyroid gland, a finding which persisted during delayed imaging at 90 minutes and 3 hours and was suggestive of right inferior parathyroid adenoma (Figure 4). The patient underwent a right inferior parathyroidectomy and a 3 X 2 X 0.5 cm parathyroid adenoma was detected. Histopathology revealed a homogenous cell population arranged in nests without any evidence of capsular or vascular invasion and absence of mitotic figures suggestive of chief cell type parathyroid adenoma (Figures 5a,5b). Calcium and calcitriol supplementation for symptomatic hypocalcemia was started on the first post-operative day. Serum intact PTH level one week post surgery was 64 pg/mL. Calcitriol was tapered and stopped 3 weeks after surgery. Monthly vitamin D supplementation along with calcium carbonate was continued. Last evaluated 4 months after surgery, the bone pains and proximal weakness had resolved, with significant improvement in the patient’s quality of life.

## DISCUSSION

Genetic causes of PHPT in children involve inactivating calcium-sensing receptor mutation. The homozygous loss of this receptor leads to severe neonatal hyperparathyroidism due to multiple gland hyperplasia, failure to thrive, respiratory distress, severe hypercalcemia, demineralization and increased mortality, whereas its heterozygous loss results in familial hypocalciuric hypercalcemia, a benign condition characterized by mild hypercalcemia (5). PHPT in children can also be due to an autosomal dominant disorder characterized by multiple gland hyperplasia, associated with multiple endocrine neoplasia (MEN)-1, hyperparathyroidism-jaw tumor syndrome, or very rarely with MEN-2 (6). In a literature review of 16 PHPT children presenting with rickets by Pitukcheewanont et al (4), genu valgum was reported as the most common presenting feature (75%), followed by rachitic rosary (50%), bone pains (37.5%), and wrist widening (31.25%). Thirteen of these children had hypercalcemia and hypophosphatemia at presentation in contrast to 3 who, as also observed in our patient, were normocalcemic at presentation, but later developed hypercalcemia following correction of the associated vitamin-D deficiency. Vitamin D has an important role in increasing calcium absorption from the gastrointestinal tract along with reducing renal loss. It can be speculated that in our patient with PHPT, deficiency of vitamin D perhaps prevented the development of hypercalcemia, which became apparent only after correction of this deficiency. PHPT in India is believed to be associated with larger parathyroid adenoma size, more severe bone disease, and as many as 50% of patients do not have hypercalcemia at initial presentation due to co-existing vitamin-D deficiency (7,8).

Our patient had several severe skeletal deformities associated with rickets, which may be explained by the synergistic effect of vitamin-D deficiency along with PHPT (7). However, typical radiological features associated with PHPT such as subperiosteal resorption, brown tumors, pathological fractures, and osteitis fibrosa cystica were lacking. Lack of sun exposure, along with nutritional deficiency, may explain the development of acquired vitamin-D deficiency. A state of malabsorption was unlikely in our patient since there were no overt gastrointestinal symptoms and no evidence of other nutrient deficiency. In spite of being a tropical country, hypovitaminosis D is extremely common among school children in India with a reported prevalence of >90% (9). Lack of clinical improvement (rickets resolution) following vitamin-D supplementation in our patient may be explained by the deleterious effect of persistently elevated PTH on chondrocytes and bone. PHPT in our patient was due to a parathyroid adenoma. In the 19 cases of PHPT with rickets reported to date, 17 were due to parathyroid adenoma, one was due to parathyroid hyperplasia, and one to an ectopic thymic parathyroid adenoma (3,4).

This case report intends to highlight that rickets can rarely be the presenting feature of PHPT. Rickets in the setting of PHPT often masks the classical clinical and biochemical phenotypic features of PHPT. In a child with rickets, lack of resolution of symptoms and signs following vitamin D supplementation, hypercalcemia at presentation, or its development following vitamin D supplementation, are warning signs for need of further evaluation to rule out PHPT.

## Figures and Tables

**Table 1:**
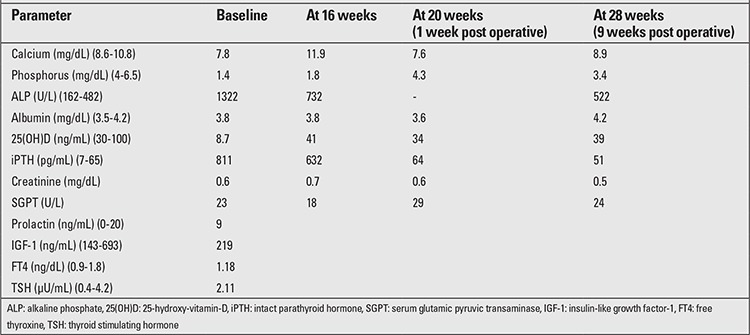
Biochemical profile of the patient during the course of her follow-up

**Figure 5a:**
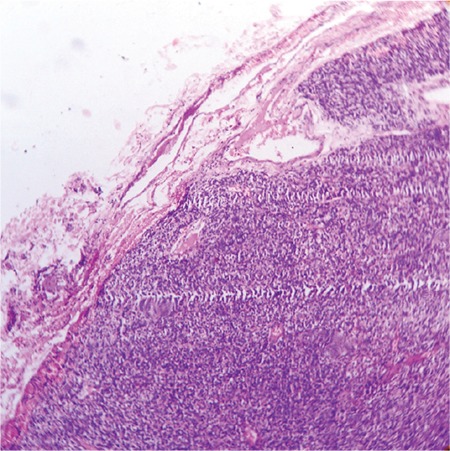
Histopathology of the surgically resected right inferior parathyroid showing sheets of cells without any evidence of capsular invasion (low magnification)

**Figure 5a:**
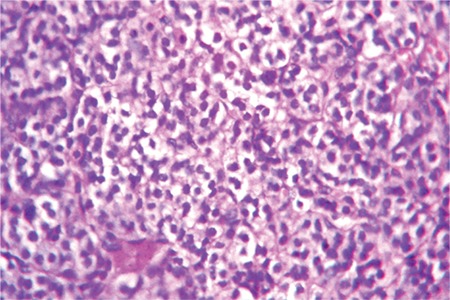
Higher magnification of the specimen showing a highly cellular homogenous cell population arranged in nests suggestive of chief cell type of parathyroid adenoma
